# Genome-wide analysis of MicroRNA-messenger RNA interactome in ex-vivo gill filaments, *Anguilla japonica*

**DOI:** 10.1186/s12864-020-6630-0

**Published:** 2020-03-04

**Authors:** Hoi Man Ng, Jeff Cheuk Hin Ho, Wenyan Nong, Jerome Ho Lam Hui, Keng Po Lai, Chris Kong Chu Wong

**Affiliations:** 1 Croucher Institute for Environmental Sciences, Department of Biology, Hong Kong Baptist University, Kowloon Tong, HKSAR Hong Kong; 2 School of Life Sciences, Simon F.S. Li Marine Science Laboratory, State Key Laboratory of Agrobiotechnology, The Chinese University of Hong Kong, Shatin, HKSAR Hong Kong; 3 Guanxi Key Laboratory of Tumor Immunology and Microenvironmental Regulation, Guilin Medical University, Huan Cheng North 2nd Road 109, Guilin, 541004 People’s Republic of China

**Keywords:** Fish gill osmoregulation, Hyperosmotic stress, Japanese eel, Transcriptome, microRNA inhibitors

## Abstract

**Background:**

Gills of euryhaline fishes possess great physiological and structural plasticity to adapt to large changes in external osmolality and to participate in ion uptake/excretion, which is essential for the re-establishment of fluid and electrolyte homeostasis. The osmoregulatory plasticity of gills provides an excellent model to study the role of microRNAs (miRs) in adaptive osmotic responses. The present study is to characterize an ex-vivo gill filament culture and using omics approach, to decipher the interaction between tonicity-responsive miRs and gene targets, in orchestrating the osmotic stress-induced responses.

**Results:**

Ex-vivo gill filament culture was exposed to Leibovitz’s L-15 medium (300 mOsmol l^− 1^) or the medium with an adjusted osmolality of 600 mOsmol l^− 1^ for 4, 8 and 24 h. Hypertonic responsive genes, including osmotic stress transcriptional factor, Na^+^/Cl^−^-taurine transporter, Na^+^/H^+^ exchange regulatory cofactor, cystic fibrosis transmembrane regulator, inward rectifying K^+^ channel, Na^+^/K^+^-ATPase, and calcium-transporting ATPase were significantly upregulated, while the hypo-osmotic gene, V-type proton ATPase was downregulated. The data illustrated that the ex-vivo gill filament culture exhibited distinctive responses to hyperosmotic challenge. In the hyperosmotic treatment, four key factors (i.e. drosha RNase III endonuclease, exportin-5, dicer ribonuclease III and argonaute-2) involved in miR biogenesis were dysregulated (*P* < 0.05). Transcriptome and miR-sequencing of gill filament samples at 4 and 8 h were conducted and two downregulated miRs, miR-29b-3p and miR-200b-3p were identified. An inhibition of miR-29b-3p and miR-200b-3p in primary gill cell culture led to an upregulation of 100 and 93 gene transcripts, respectively. Commonly upregulated gene transcripts from the hyperosmotic experiments and miR-inhibition studies, were overlaid, in which two miR-29b-3p target-genes [Krueppel-like factor 4 (klf4), Homeobox protein Meis2] and one miR-200b-3p target-gene (slc17a5) were identified. Integrated miR-mRNA-omics analysis revealed the specific binding of miR-29b-3p on Klf4 and miR-200b-3p on slc17a5. The target-genes are known to regulate differentiation of gill ionocytes and cellular osmolality.

**Conclusions:**

In this study, we have characterized the hypo-osmoregulatory responses and unraveled the modulation of miR-biogenesis factors/the dysregulation of miRs, using ex-vivo gill filament culture. MicroRNA-messenger RNA interactome analysis of miR-29b-3p and miR-200b-3p revealed the gene targets are essential for osmotic stress responses.

## Background

The catadromous fish Japanese eels have a complex life cycle in freshwater and seawater environments. The fish is euryhaline and actively engage physiological responses to oppose osmotic perturbations to stabilize body osmolality. Fish gills therefore possess great structural and functional plasticity in response to variations of water osmolality, to support the process of fluid and electrolyte homeostasis [1, 2]. In response to osmotic stress, a distinct suite of modifications in gill epithelia is activated for functional adaptation, which involves cell proliferation and differentiation, changes in the activities, expressions and trafficking of different ion transporters/channels [3, 4]. In the past decades, considerable numbers of reports identified the underlying molecular and physiological factors associated with ion-osmoregulatory functions of gills. In recent, advances in the understanding of small non-coding RNAs such as microRNAs (miRs) on regulatory circuit in physiological and pathophysiological functions shed light on roles of miRs in osmotic stress responses.

In mammals, the involvement of miRs in fluid and electrolyte transport has become apparent. A number of studies have illustrated the implications of miRs in ion transport functions. For instances, a direct regulatory role of miR-9 in the expression of large-conductance calcium and voltage-activated potassium channels in neuronal ion transport in alcohol tolerance phenomenon in an adult rat model was identified [5]. The involvement of miR-133a and miR-133b in primary culture of canine cardiomyocytes [6] and miR-1 [7] on potassium channels for cardiac arrhythmias in human samples and rat models were demonstrated. Moreover, indirect regulatory roles of miRs (i.e. miR-155, miR124 & miR-135a) on ion transport were reported via their inhibitory actions on receptors of electrolyte-regulated hormones, angiotensin-I receptor or mineralocorticoid receptor, in screening samples of patients with hypertension [8, 9]. The expression level of the with-no-lysine kinase (*wnk1*, a regulator of electrolyte homeostasis) in mouse nephrons, was found to be regulated by miR-192. Its expression was modulated by physiological stimuli (i.e. aldosterone or salts loading) [10]. In renal medullary epithelial mIMCD3 cells, tonicity was also identified as a stimulus to regulate miR-200b and miR-717 expression, which inhibit the expression of the transcriptional factor, osmotic response element binding protein (*orebp*) [11]. In comparison, studies of miRs in osmotic stress responses in fish are limited. Using zebrafish embryos, the expression of the miR-8 family (miR-200) in ionocytes was found to inhibit the expression of Na^+^/K^+^-exchanger regulatory cofactor (*nherf1*) to impair cellular responses to osmotic stress [12]. Using tilapia, decreased expression levels of miR-429 in gills under hyperosmotic stress was observed. An in-vivo inhibitory effect of miR-429 on the expression of osmoregulatory transcription factor (*ostf1*) in gills [13] and an inhibitory action of miR-30c on the expression of renal hsp70 under hyperosmotic stress [14] were reported. Moreover, differential expression patterns of miRs in gills of marbled eels, *Anguilla marmorata* adapted at different salinities [i.e. freshwater (FW), brackish-water or seawater (SW)] were described [15]. Japanese eels are euryhaline fish. The osmoregulatory tissue - gills provides an excellent model to study role of miRs in the regulation on plasticity of adaptive osmotic responses in vertebrates. The present study aimed to identify and characterize the involvement of miRs and messenger RNAs (mRNA) under osmotic perturbations. In this report, we used both ex-vivo gill filament and primary gill cell culture models, accompanied with miR-, transcriptome-sequencing and miR inhibition, to identify tonicity-sensitive miRs and to characterize their expressions in gill filaments.

## Results

### Hyperosmotic treatment induced differential expression of transcriptional Factor-1/regulators, ion channels/transporters and miR biogenesis factors in ex-vivo gill filament culture

**Fig. 1:**
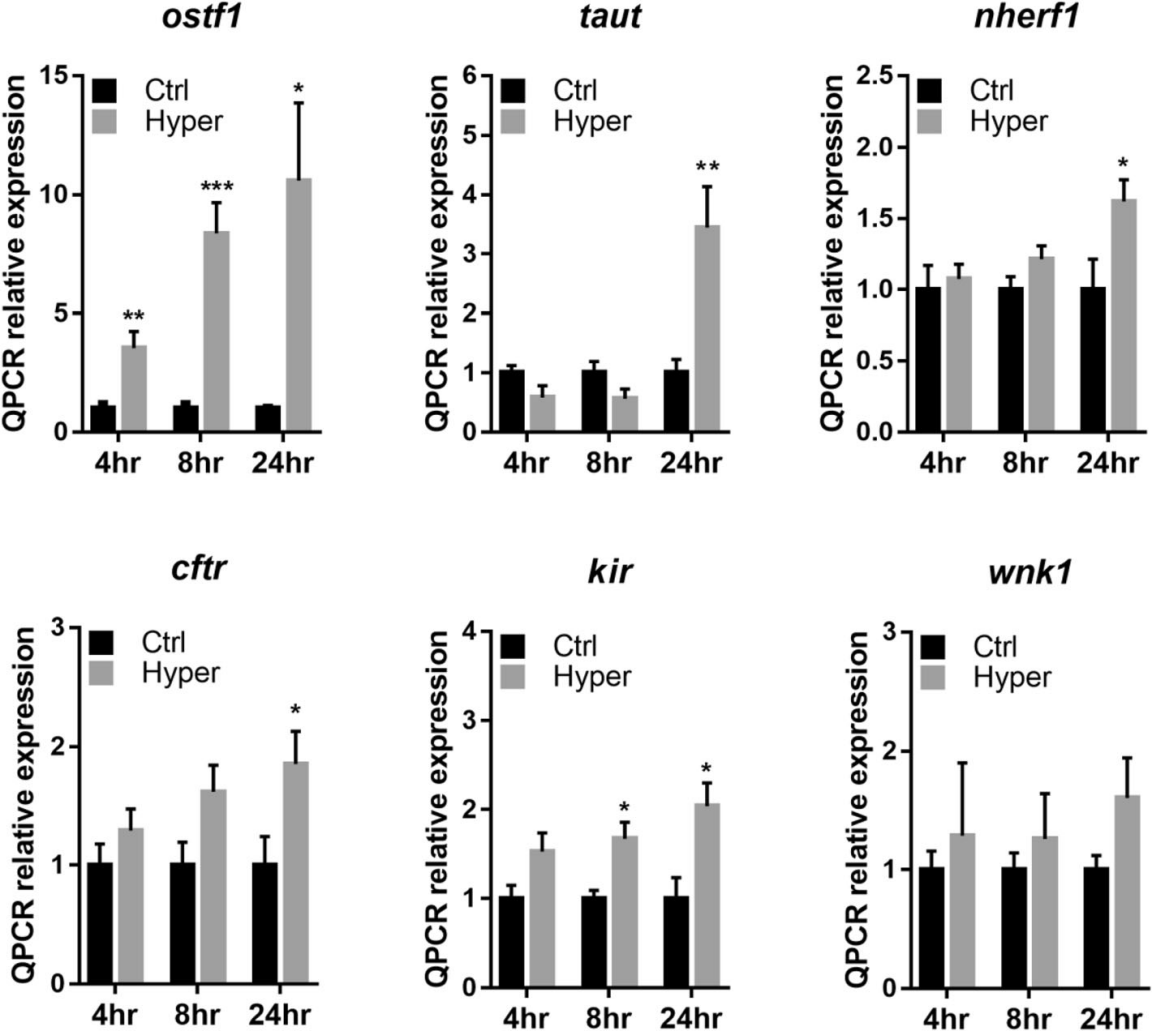
Hypertonic stress induces differential gene expression of osmotic stress transcriptional factor and seawater ion transporter. Eel gill filaments were challenged in hyperosmotic (Hyper: 600 mOsmol l^− 1^) media for 4, 8 and 24 h. Control (Ctrl: 300 mOsmol l^− 1^) or hypertonic-treated gill filaments were subsequently processed for analysis of tonicity-responsive gene expression by quantitative SYBR Green Real-Time PCR (qRT-PCR). Differential gene expression of osmotic-stress transcriptional factor (*ostf1*), Na^+^/K^+^ exchange regulatory cofactor (*nherf1*), with-no-lysine kinase 1 (*wnk1*), inward rectifying K^+^ channel (*kir*), cystic fibrosis transmembrane conductance regulator (*cftr*) and Na^+^/Cl^−^-taurine transporter (*taut*) was determined. *Gapdh* expression was used as internal control for normalization of target gene expression. Results (mean ± s.e.m.) were from five independent experiments. * *P* < 0.05, ** *P* < 0.01, *** *P* < 0.001 vs control

**Fig. 2:**
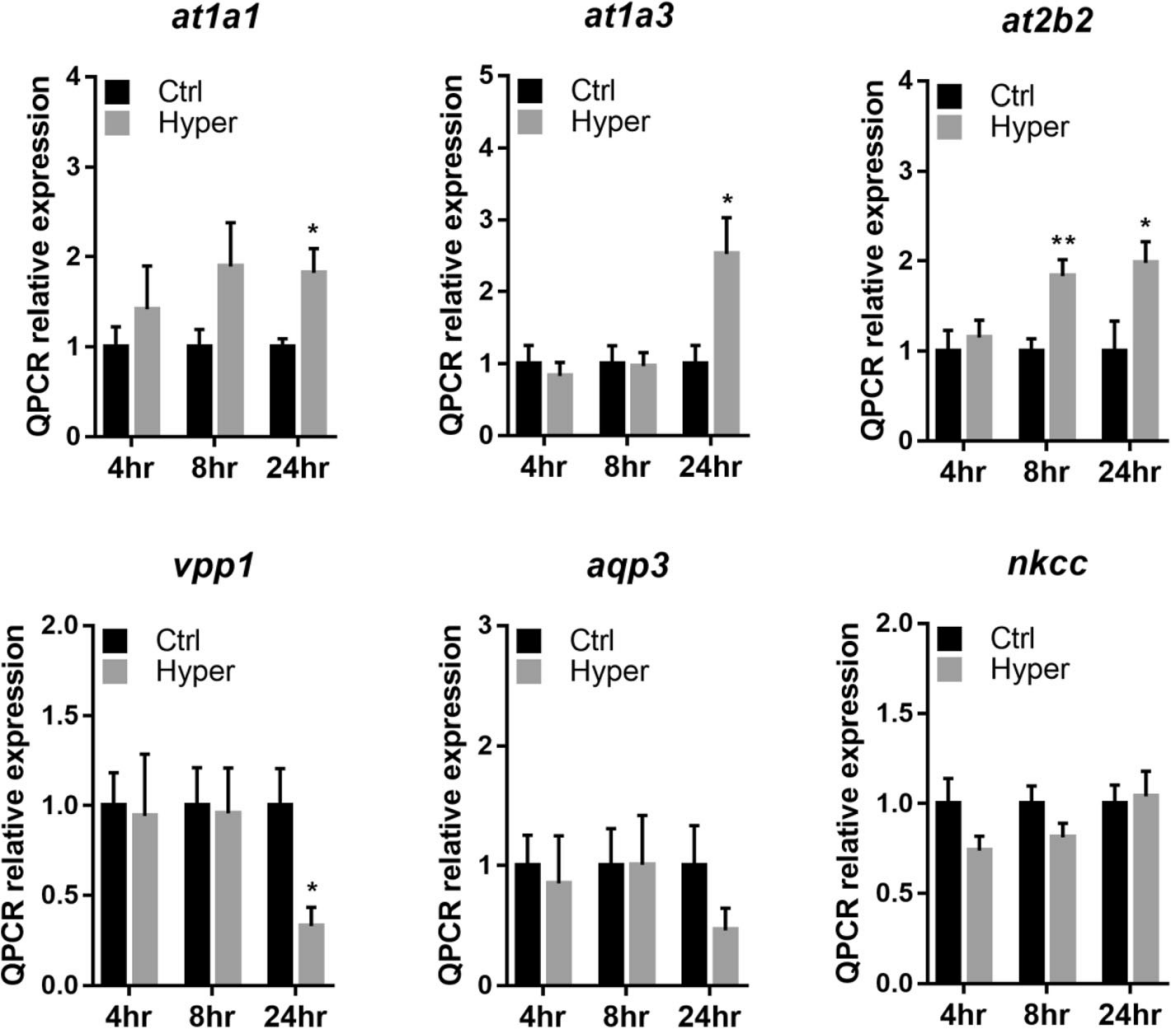
Hypertonic stress induces differential gene expression of ion transporting enzyme in gill filament culture. Differential gene expression of ATPase Na^+^/K^+^-transporting subunit alpha 1 (*at1a1*), ATPase Na^+^/K^+^-transporting Subunit Alpha 3 (*at1a3*), calcium-transporting ATPase 2 (*at2b2*), aquaporin 3 (*aqp3*), V-type proton ATPase 1 (*vpp1*) and Na^+/^K^+^/2Cl^−^-cotransporter (*nkcc*) was determined in control (Ctrl: 300 mOsmol l^− 1^) or hypertonic-treated gill filaments ((Hyper: 600 mOsmol l^− 1^, 4, 8, 24 h) by qRT-PCR. Relative expression level was normalized by *gapdh*. Results (mean ± s.e.m.) were from five independent experiments. * *P* < 0.05, ** *P* < 0.01 vs control

Gill filaments of eels were ex-vivo cultured in isotonic (300 mOsmol l^− 1^) or hypertonic (600 mOsmol l^− 1^) L-15 medium (penicillin-streptomycin (PS), gentamycin, FBS) at 23 °C for 4, 8 and 24 h. Transcripts levels of *ostf1*, Na^+^/Cl^−^-taurine transporter (*taut*), Na^+^/H^+^ exchange regulatory cofactor (*nherf1*), cystic fibrosis transmembrane regulator (*cftr*), and inward rectifying K^+^ channel (*kir*), were significantly upregulated at hypertonic medium, in a time-dependent manner (Fig. 1). Moreover, the adenosine triphosphatases (ATPases), including the subunits of Na^+^/K^+^-ATPase [ATPase Na^+^/K^+^ transporting subunit α1 (*at1α1*), ATPase Na^+^/K^+^ transporting subunit α3 (*at1α3*)], and calcium-transporting ATPase 2 (*at2b2*) were increased (*p* < 0.05). The mRNA expression level of V-type proton ATPase 1 (*vpp1*) was significantly reduced (*p* < 0.05) while the levels of aquaporin-3 (*aqp-3*) and Na^+^/K^+^/2Cl^−^ cotransporter (*nkcc*, *s12a2*) mRNA showed no noticeable changes (Fig. 2).

**Fig. 3:**
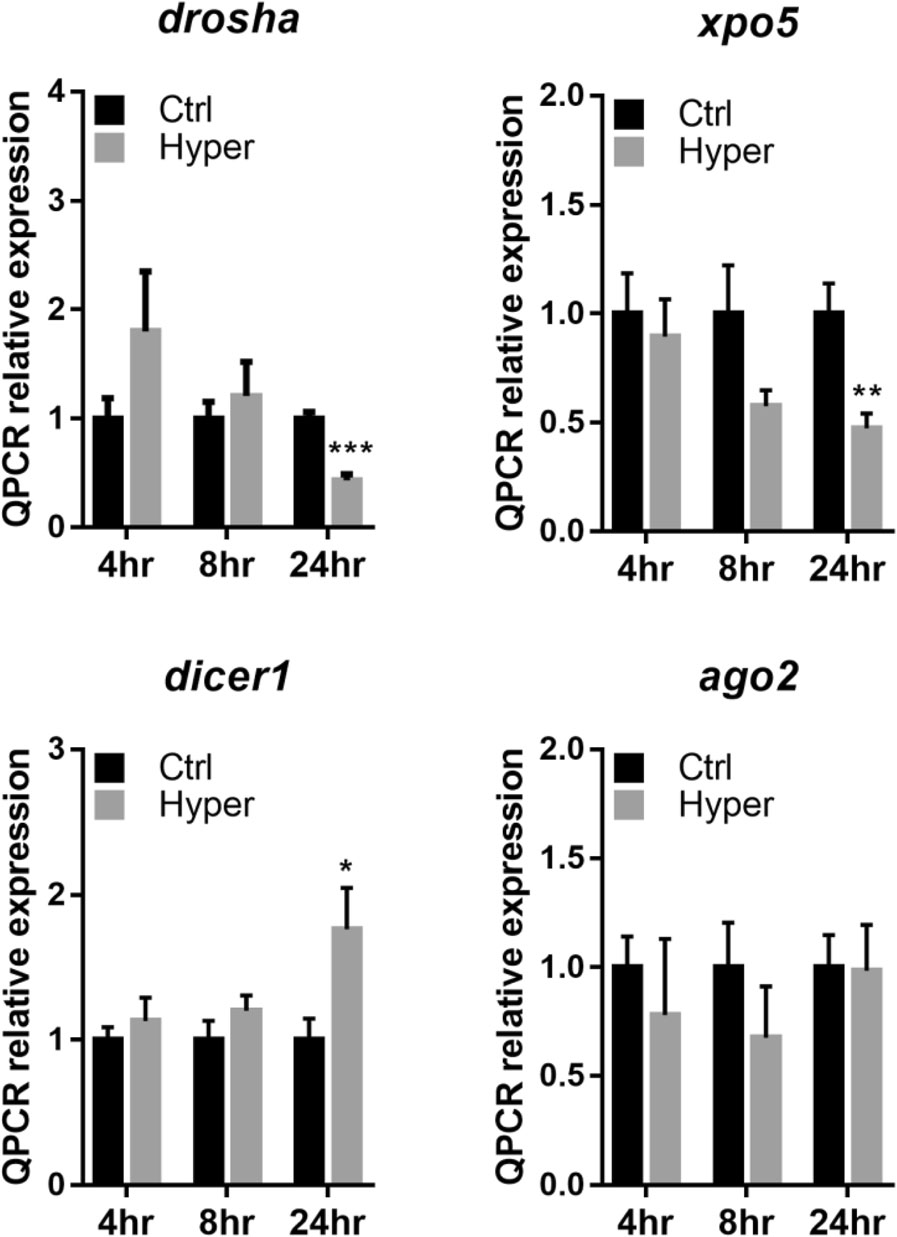
Hypertonic stress limits gene expression of miRNA biogenesis factors in gill filament. Gill filaments were subjected to different time-point of hypertonic treatment in the differential gene expression analysis. Expression of miRNA biogenesis key factors, drosha, exportin 5 (*xpo5*), dicer1 and argonaute 2 (*ago2*), was determined in control (Ctrl: 300 mOsmol l^− 1^) or hypertonic-treated (Hyper: 600 mOsmol l^− 1^) gill filaments for 4, 8 and 24 h by qRT-PCR. *Gapdh* was used as internal control. Results (mean ± s.e.m.) were from five independent experiments. * *P* < 0.05, ** *P* < 0.01, *** *P* < 0.001 vs control

For the key factors involved in miR biogenesis, the mRNA expression levels of drosha RNase III endonuclease (*drosha*), exportin-5 (*xpo5*), dicer ribonuclease III (*dicer-1*) and argonaute-2 (*ago2*) in ex-vivo gill filaments were measured at 4, 8 and 24 h of post-hypertonic treatment (Fig. 3). In general, the expression levels of *drosha*, and *xpo5* transcripts were significantly reduced under hyperosmotic stress. The expression levels of *dicer-1*, however was significantly increased.

### Hyperosmotic treatment induced differential expression of miRs in ex-vivo gill filament model

**Fig. 4:**
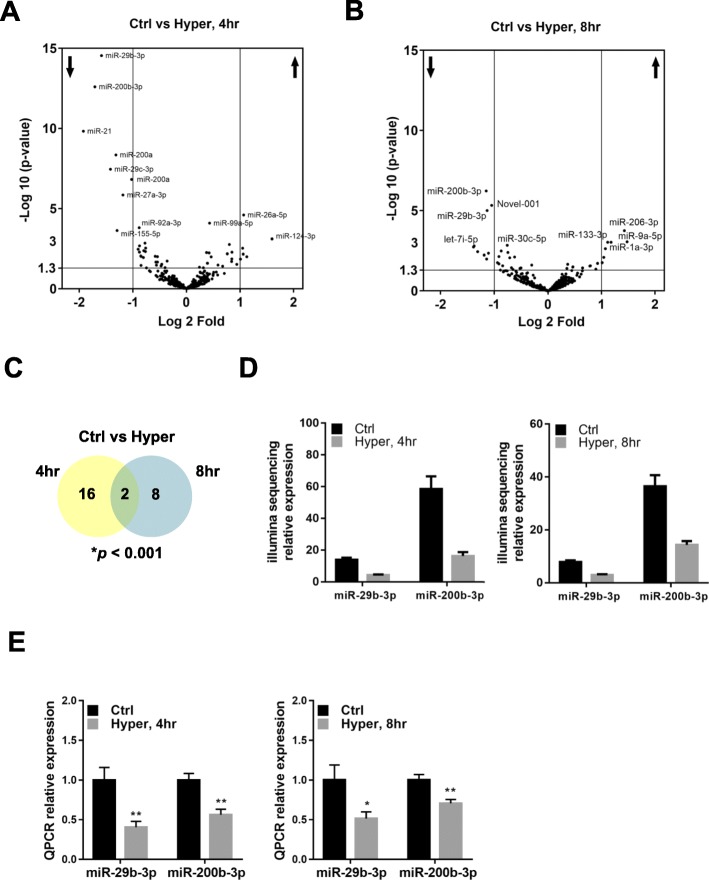
Expression of miR-29 and miR-200 is dysregulated in gill filament culture under hypertonic stress. Gill filaments were challenged in hyperosmotic (Hyper: 600 mOsmol l^− 1^) media for 4 h and 8 h and were subsequently processed for small RNA sequencing. **a** & **b** Volcano plot showed differentially expressed miRs under 4 h (**a**) or 8 h (**b**) hypertonic treatment in gill filaments compared with the control. The *p* < 0.05 and |Log2 (fold change)| > 1 were set as threshold for significantly differential expression. In 4 h hypertonic treatment, 81 differentially expressed miRs including 42 up-regulated miRs and 39 down-regulated miRs were identified, while in 8 h hypertonic treatment, 55 differentially expressed miRs including 24 up-regulated miRs and 31 down-regulated miRs were identified. **c** Venn diagram shows the similarity of differentially expressed miRs under 4 h and 8 h hypertonic treatments. Two miRs, miR-29b-3p and miR-200b-3p, with significant expression changes (*p* < 0.001) in both hypertonic treatment groups were selected for further analysis. **d** Bar charts exhibit the differential expression levels of miR-29b-3p and miR-200b-3p in control and hypertonic groups (4 or 8 h) based on deep sequencing data. **e** qRT-PCR was performed to validate the expression pattern of miR-29b-3p and miR-200b-3p. snRNA U6 was used as internal control for normalization of miRNA expression. Results (mean ± s.e.m.) were from five independent experiments. * *P* < 0.05, ** *P* < 0.01 vs control

Gill filaments of eels were ex-vivo cultured in isotonic (300 mOsmol l^− 1^) or hypertonic (600 mOsmol l^− 1^) L-15 medium (PS, gentamycin, FBS) at 23 °C for 4 and 8 h. Total RNA was extracted and isolated using mirVana (Invitrogen). The A_260_/A_280_ value of the isolated RNA was > 1.8, and the RIN was over 8. Library construction was prepared for miR sequencing. A total of 568 million quality-trimmed raw reads were obtained from the small RNA sequencing (Additional file 1: ST1). De novo analysis identified 658 and 662 miR precursors from the sequencing samples at 4 and 8 h respectively (Table 1). In consideration of Randford *p*-value, there were 82 and 84 miR precursors found to be not significant at 4 and 8 h treatment respectively. The corrected miR precursors were 576 and 578 at 4 and 8 h treatments. Figures 4a, b show the volcanic plots of deregulated miRs of the samples. In the 4 h treatment, the expression levels of 81 miR precursors were found to be significantly different (DESeq2 adjusted *p*-value < 0.05). There were 42 miRs upregulated and 39 miRs downregulated. In 8 h treatment, there were 55 differentially expressed miR precursors in which 24 miR precursors were upregulated and 31 were downregulated (DESeq2 adjusted *p* < 0.05). To prioritize potential differentially regulated miRs, the significance of *p*-value was set to *p* < 0.001. This led to a reduction in the numbers of differentially expressed miRs from 81 to 18 at 4 h, and 79 to 10 at 8 h treatments. The Venn diagram (Fig. 4c) shows the common differentially expressed miRs at 4 and 8 h. The two miRs, miR-29b-3p and miR-200b-3p were commonly downregulated at both time points of the hyperosmotic treatments (Fig. 4d). The differentially expressed miRs were validated using real-time PCR of the samples from isotonic and hypertonic treated gill filaments (Fig. 4e).

**Table 1:** Number of dysregulated miRNA caused by hypertonic stress

ex vivo ***approach***	small RNA seq *n* = 5		Japanese eel gill filaments	
**600 mOsmol l-1 4 h**				
Ctrl vs Hyper 600 mOsmol l-1 4 h	***p-value*** **< 0.05**	***p-value*** **> 0.05**	***p-value > 1***	**Total**
All miRNA precursors	101	512	45	658
Randford *p*-value (not significant)	20	58	4	82
Removed miRNA precursor with not significant Randford *p*-value				658–82
				576
	***p-value*** **< 0.05**	***p-value*** **> 0.05**	***p-value > 1***	**Total**
Ctrl vs Hyper 600 mOsmol l-1 4 h	81	454	41	576
up regulated	42	255	19	316
down regulated	39	199	22	260
**600 mOsmol l-1 8 h**				
Ctrl vs Hyper 600 mOsmol l-1 8 h	***p-value*** **< 0.05**	***p-value*** **> 0.05**	***p-value > 1***	**Total**
All miRNA precursors	65	556	41	662
Randford *p*-value (not significant)	10	69	5	84
Removed miRNA precursor with not significant Randford *p*-value				662–84
				578
	***p-value*** **< 0.05**	***p-value*** **> 0.05**	***p-value > 1***	**Total**
Ctrl vs Hyper 600 mOsmol l-1 8 h	55	487	36	578
Up-regulated	24	282	19	325
Down-regulated	31	205	17	253

**Fig. 5:**
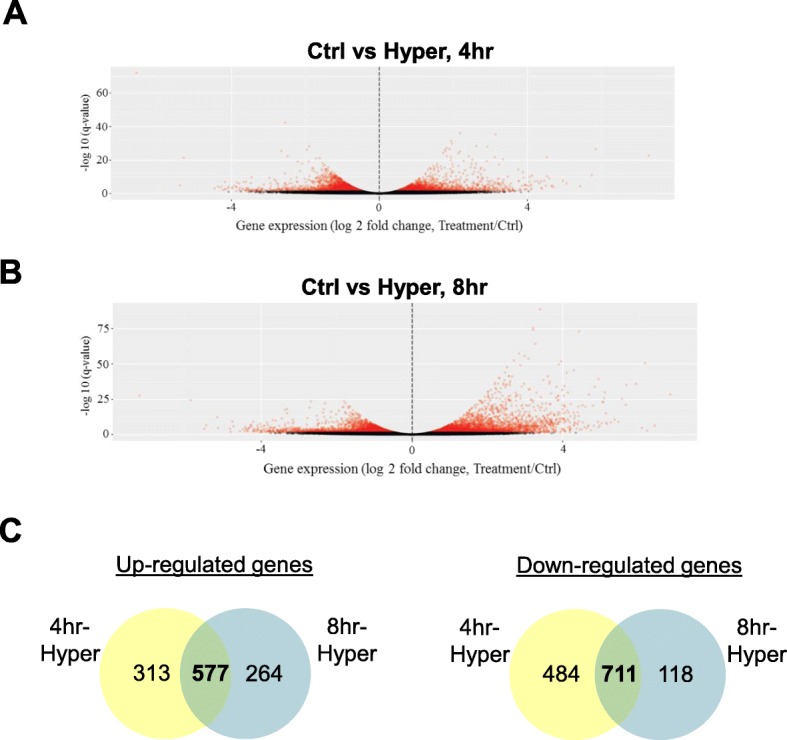
Hypertonic stress mediates differential gene expression in gill filament culture. Total RNA of gill filaments with small RNA sequencing were subjected for transcriptome analysis (*n* = 5). **a** & **b** Volcano plot showed DEGs under 4 h (**a**) or 8 h (**b**) hypertonic treatment (Hyper: 600 mOsmol l^− 1^) as compared to the control (Ctrl: 300 mOsmol l^− 1^). DEGs with |log2 (fold change)| > 1, −log10 *q*-value > 2 were set as threshold for significantly differential expression. In 4 h hypertonic treatment, 2085 DEGs including 890 upregulated genes and 1195 downregulated genes were identified, while in 8 h hypertonic treatment, 1670 DEGs including 841 upregulated genes and 829 downregulated genes were identified. **c** Venn diagram shows 577 commonly upregulated or 711 downregulated genes between 4 h and 8 h hypertonic treatment groups

Since the suppression of gene expression is one of the major functions of miR, a transcriptome analysis of differential gene expression in ex-vivo gill filament-culture was conducted. There were 2085 differentially expressed genes (DEGs) at 4 h of the hypertonic treatment (Fig. 5a), including 890 upregulated genes and 1195 downregulated genes (Additional file 1: ST3). In 8 h of hyperosmotic treatment, 1670 DEGs including 841 upregulated genes and 829 downregulated genes were identified (Fig. 5b, Additional file 1: ST4). In the comparison of DEGs from the 4 h and 8 h treatments, 577 commonly upregulated and 711 downregulated genes were observed (Fig. 5c).

### Integrated miR-inhibition and Transcriptome analysis in primary gill culture model

**Fig. 6:**
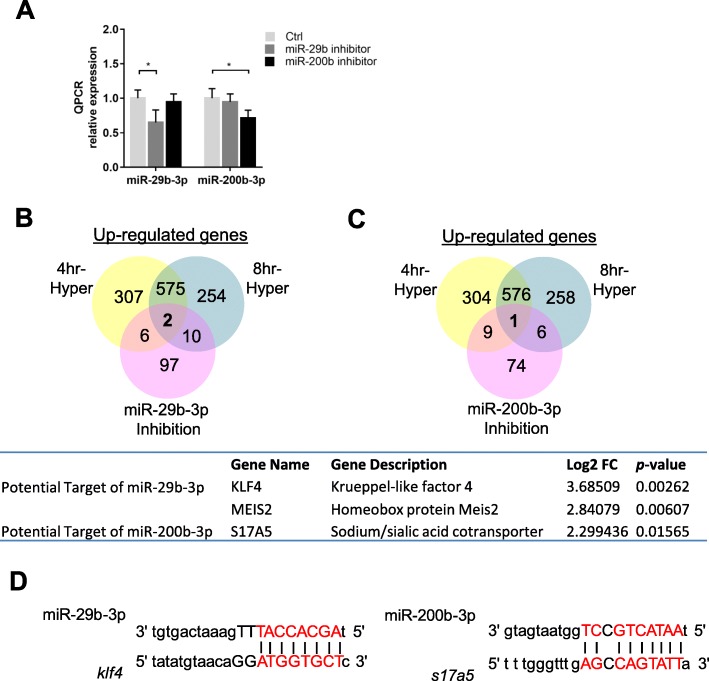
Inhibition miR-29b-3p/miR-200b-3p in primary gill cell culture. To determine the biological effects of miR-29b-3p and miR-200b-3p through loss-of-function, two specific miRNA inhibitors (miR-29b-3p or miR-200b-3p) were transfected in gill cell culture. **a** qRT-PCR was performed to determine the efficiency of inhibition on miR-29b-3p and miR-200b-3p. Venn diagram shows the overlay of up-regulated genes in 4/8-h hypertonic (Hyper) treatment and **b** miR-29b-3p inhibition, and **c** miR200b-3p. The figures in parentheses represent the numbers of co-expressed genes. The potential target genes of miR-29b-3p and miR-200-3p were listed in the table. **d** Using miRanda algorithm, the full length of the miR was aligned against the target gene sequences. Thermodynamic stability of the RNA duplexes is based on these alignments and strict alignment in the seed region at position 2–7 bp of the miR to the mRNA sequence. * *P* < 0.05 vs control

To underpin the gene targets, specific miR-inhibitors were used to block the activities of miR-29b-3p and miR-200b-3p. Figure 6a showed the effects of the inhibition on the individual miRs in primary gill cell culture. The inhibition of miR-29b-3p did not affect the expression level of miR-200b-3p, and vice versa. Supplementary Tables 5 and 6 showed the list of upregulated gene targets in cells after the treatment with miR-29b-3p and miR-200b-3p inhibitors, respectively. We overlaid the transcriptomic data of 4- and 8 h-upregulated genes (ST3 & ST4) and of miR-inhibition, to select two target-genes [i.e., krueppel-like factor 4 (klf4) and homeobox protein meis2] for miR-29b-3p (Fig. 6b), and one target-gene (slc17a5) for miR-200b-3p (Fig. 6c). miRanda algorithm was then used to predict the binding of the individual miRs to the target genes. TransDecoder (version 5.0.2) was used to determine the coding regions including 5′ UTRs and 3′ UTRs of transcripts [16]. The analysis showed that miR-29b-3p and miR-200b-3p could bind to the 3′ untranslated region (3’UTR) of *klf4* and slc*17a5* (Fig. 6d). The binding site of *klf4* is at 3’UTR between 447 to 468, while the binding site of *s17a5* is at the 3’UTR between 53 to 73.

## Discussion

miRs are a class of endogenous and conserved small RNA molecules (~ 22 nt) that play gene regulatory roles in targeting mRNAs for cleavage or translational repression [17]. Based on the finding of the recent studies, miRs are found to have a pivotal role in the regulation of fluid and electrolyte balance [18, 19]. It warrants further investigation to identify tonicity-inducible miRs and to determine their potential roles in osmoregulatory responses. In the present study, using ex-vivo gill filament culture model, we characterized the hyperosmotic responses with respective to the expression levels of some well-defined hyper- and hypo-osmotic genes (i.e. regulators and transporters). The involvement of miR-biogenesis genes and tonicity-responsive miRs were characterized. Possible mRNA candidates were predicted using genome-wide analysis of microRNA-mRNA interactome.

In the first part of the study, we characterized the ex-vivo gill filament culture model with regard to its responses to hyper-osmotic stress. The purpose is to establish a culture model, which retains the three-dimensional organization (primary and secondary lamellae) and takes into account of all the cell types [pavement cells (PVCs), chloride cells (CCs), mucous cells and undifferentiated cells] of gill tissues in experiments. In this study, numerous well-characterized hyper-osmotic and hypo-osmotic inducible genes were chosen to evaluate the functional responses and the validity of the culture. The hyperosmotic inducible genes, like *ostf1* [20, 21], *nherf* [22], *wnk1* [23] are regulatory proteins known to modulate gene transcription, apical trafficking of transmembrane G-protein coupled receptors/ion-transporters and epithelial chloride (Cl^−^) transport respectively. The hyperosmotic *ostf1* induction was demonstrated in intact fish and primary gill epithelial cell culture [24]. Its expression could also be stimulated by the seawater-adapting hormone, cortisol [25]. The orchestrating role of ostf1 to integrate environmental and hormonal signals for osmosensory function was documented. In our study, *ostf1* mRNA was stimulated in our ex-vivo culture model, under hyperosmotic stress. Our data also showed the upregulation of *nherf1*, suggesting its function in hyperosmotic adaptation. Intriguingly, it was reported that nherf1 negatively regulated the apical localization of Na^+^/H^+^ exchanger (*nhe*) in renal brush border cells [26]. *Nhe* facilitates Na^+^ retention in hypotonic solution, in exchange of H^+^, the process is important for freshwater adaptation. *Nhef1* was found to be expressed in ionocytes in zebrafish embryos [12]. The downregulation of *nherf1* blocked Na^+^ accumulation in ionocytes. Presumably, in our study the upregulation of *nherf1* in hyperosmotic gill filaments might downregulate *nhe*. Hyperosmotic acclimation is known to stimulate Na^+^ and Cl^−^ secretion in ionocytes via the upregulation of *kir*, Na^+^/K^+^-ATPase (*at1a1* and *at1a3*), and *cftr*. The sodium pump generated an extracellular Na^+^ gradient to facilitate the transcellular co-transport of Cl^−^ to cell cytoplasm, followed by Cl^−^ secretion via apical *cftr*. All these transporters were upregulated in the hyperosmotic treated gill filaments. The calcium-transporting ATPase 2 (*at2b2*) is known to reduce intracellular Ca^2+^ to maintain calcium homeostasis, which could be perturbed by osmotic stress and seawater adaptation in eels [27, 28]. The Na^+^/K^+^/2Cl^−^-cotransporter (*s12a2*) aids in the secondary transport of Na^+^, K^+^ and Cl^−^, driven by Na^+^-gradient established by Na^+^/K^+^-ATPase [29]. However, there was no noticeable induction of *sl2a2* in this culture model. In addition to ion secretion, hyperosmotic acclimation involves the accumulation of organic osmolytes, like taurine to increase cellular osmolality. The expression levels of the taurine transporter, taut was upregulated in the hyperosmotic treated gill filaments. The aquaporin-3 (*aqp-3*) that mediates the translocation of water, glycerol, urea and other small solutes, was highly expressed in freshwater eel gills [30]. No significant reduction in its expression levels was measured in this study. On the other hand, the transporter (*vpp1*) involved in hypo-osmotic acclimation, was downregulated in the hyperosmotic treated gill filaments. As compared with the primary gill cell model, it is a two-dimensional culture and comprises mostly PVCs [31, 32]. In the past studies, using primary gill PVC culture under hyperosmotic treatment, upregulation of *ostf1*, *Na*^*+*^*/K*^*+*^*-ATPase* and *taut* mRNA expression were detected [31, 33]. The stimulation of *cftr, kir, and nherf1* expression in the hyperosmotic-treated culture, has not yet been reported. Indeed, the expression of *cftr* and *kir* are known to be cell-specific, localized in seawater CCs. The low percentage of CCs in the primary cell culture made the model not representative as compared with the ex-vivo gill filament culture for studying gill physiology. Collectively, our data demonstrated that the ex-vivo gill filament culture exhibited distinctive responses to hyperosmotic acclimation. It would be a useful model to investigate underlying mechanisms of osmotic responses in fish gills.

With hindsight, the involvement of miRs in fluid and electrolyte transport has become apparent. A number of studies have illustrated the implications of miRs in ion transport in renal and non-renal tissues, showing a pivotal role of miRs in the regulation of fluid and electrolyte balance. In the present study, we studied the expression of four major factors involved in miR biogenesis, using the ex-vivo gill filament culture. Briefly, RNA polymerases II and III are involved in primary-miR (pri-miR) transcription [34, 35]. Upon nuclear cleavage of pri-miR by the Drosha RNase III endonuclease, a ~ 60-70 nt miRNA precursor (pre-miRNA, a stem loop intermediate) is produced and transported from cell nucleus to cytoplasm in a Ran-GTPase dependent manner by the export receptor Exportin-5 [36, 37]. The pre-miRNA is then processed in the cytoplasm by the action of another RNase III endonuclease, Dicer to produce mature miR, followed by Argonaut proteins to target through sequence complementarity in RNA-induced silencing complex [38]. In this study, the mRNA expression levels of *drosha, xpo5* and *dicer1* were dysregulated under hyperosmotic treatment, indicating that the treatment modulated miR-biogenesis in gill cells. Since miRs take part in various aspects of physiological functions, including tissue remodeling and cell survival, it is anticipated an alternation of miR expression in the hyperosmotic treated gill filaments. Thus, miR sequencing was conducted using the gill filaments at two consecutive time-points. Bioinformatics analysis of the dysregulated miRs from the two time-points at high stringency of filtering (*p* < 0.001) shortlisted two miR candidates, miR-29b-3p and miR-200b-3p. In the literatures, the family of miR-29 was found to respond to environmental stress and cellular repairing processes. In zebrafish model, the involvement of miR-29 in acute environmental stress (cold stress) was studied [39], in which the miR targeted on a core clock gene per2 to enhance cold tolerance of the fish larvae. Moreover, miR-29b was reported to play a role in cell regeneration, associated with the processes of cell survival and cytoskeleton reorganization in zebrafish [40]. Furthermore, miR-29 was recognized to modulate iron transport and oxidative stress in neurons of killifish brain [41]. Likewise, miR-200 family comprises of the -a, −b and -c members, those were reported to regulate somatic growth and neurogenesis in zebrafish [42, 43]. A study showed the involvement of miR-200 in growth hormone (GH)/insulin-like growth factor (IGF)-signaling pathway and a high expression level of miR-200 was associated with p53 to induce apoptosis [43]. The similar functional association was found to play roles in sperm motility of zebrafish [44]. Intriguingly, miR-200 was involved in epithelial ion transport to inhibit the expression of nherf1 in ionocytes of zebrafish embryos [12]. Our data agreed with the study to show that the downregulation of miR-200b-3p was accompanied with an upregulation of nherf1 in hyperosmotic gill filaments. Nonetheless, immunological function of miR-200 on the regulation of Toll-like receptor pathways in sea cucumber was described [45].

In the last part of this study, we integrated the transcriptome data of time-course hyperosmotic treatment and of RNA inhibition, followed by miRanda algorithm analysis to determine the miR-mRNA binding. *Klf4* and *slc17a5* were identified to be the target-genes of miR-29b-3p and miR-200b-3p, respectively. *Klf4* is a zinc-finger transcriptional factor essential for terminal differentiation of intestinal epithelium and skin epidermis [46, 47]. A recent study demonstrated the role of *Klf4* in the maintenance of ionocyte progenitor’s population in zebrafish embryos [48]. Chloride cells in fish gills are ionocytes responsible for ion transport. During the course of seawater acclimation, there are significant increase of chloride cells in fish gills for hypo-osmotic adaptation [49]. Presumably, in this study, the hyperosmotic treatment of gill filament culture induced the expression of *klf4* to increase chloride cell densities. The miR-200b-3p target-gene, *slc17a5* belongs to the solute carrier family of transporters to transport amino acids [50], which can serve as organic osmolytes to regulate cellular osmolality, against hyperosmotic stress [51]. The implication of the data are consistent with the physiological responses of fish gills.

## Conclusions

This is the first study to characterize the hypo-osmotic responses, and the dysregulation of miR-biogenesis factors/miRs in ex-vivo gill filament culture under hyperosmotic challenge. The data showed the culture retained the in-vivo molecular responses to upregulate hyper-osmotic factors/transporters and to downregulate hypo-osmotic transporter. Using multiple experimental approaches, two miRs and two target genes were identified to be involved in the hyperosmotic challenge of gill filaments. The two miRs were predicted to target a cluster of genes involved in osmoregulation. MiR inhibition analysis further identified two gene targets, those are essential for differentiation of gill chloride cells and the regulation of cellular osmolality. There are many other tonicity-responsive miRs and genes were identified in this study. The data support further analysis of other miRs and transcripts to elucidate their regulatory roles in osmotic responses.

## Methods

### Fish maintenance, in-vitro and ex-vivo gill culture model

Japanese eels were obtained from fish vendor Lok Fu market. Japanese eels (600-800 g) was reared in fiberglass tanks supplied with charcoal-filtering aerated freshwater water at 18–20 °C under a 12 L:12D photoperiod for at least 2 weeks to facilitate acclimation before experiments. The eel was handled in accordance to the guidelines and regulation of Hong Kong Baptist University. The fish were anesthetized with 0.1% MS-222 (Sigma) and perfused with phosphate buffered saline (PBS, pH 7.7). Gill tissue was excised and washed for the procedures of ex-vivo gill filament culture. Briefly, gill arches were cut every 5 mm interval with intact primary filaments, washed with PBS, pH 7.7 and maintained in Leibovitz’s L-15 medium (Gibco, Invitrogen, Grand Island, NY, USA) supplemented with 10% fetal bovine serum (FBS, HyClone®, Perbio Sciences, Logan, UT, USA), 1% penicillin/streptomycin, 0.5% fungizone (Gibco, Invitrogen). The culture was incubated at 23 °C.

For primary gill cell culture, the gill filaments were then subject to hyperosmotic-treatment. For primary gill cell culture, gill filaments were cut into small fragments and subjected to two cycles of tryptic digestion (0.5% trypsin + 5.3 mmol l^− 1^ EDTA) (Sigma), each for 20 min at room temperature in a rotator (300 r.p.m.). Cell suspension was filtered through stainless steel mesh (104 and 73.7 μm, Sigma), washed with PBS, pH 7.7 and finally resuspended in Leibovitz’s L-15 medium (Gibco, Invitrogen, Grand Island, NY, USA) supplemented with 10% fetal bovine serum (FBS, HyClone®, Perbio Sciences, Logan, UT, USA), 1% penicillin/streptomycin, 0.5% fungizone (Gibco, Invitrogen) and seeded at a density of 2 × 10^6^ cells cm^− 2^ onto collagen coated culture plates (Iwaki, Chiba, Japan). The culture was incubated at 23 °C in a growth chamber with humidified air. One day after seeding, each culture well was rinsed with PBS (pH 7.7) to remove mucous and unattached cells. The culture was subject to miR-knockdown analysis.

### Hyperosmotic challenge and RNA preparation

Hypertonic medium was prepared by an addition of 3% of 5 M NaCl to the Leibovitz’s L-15 medium (300 mOsmol l^− 1^). The osmolality of freshly prepared hypertonic media was measured by a vapor pressure osmometer (Wescor, 5500XR, Logan, UT, USA). For the gill filament culture, tissue samples were exposed to the Leibovitz’s L-15 medium (300 mOsmol l^− 1^) or the medium with an adjusted osmolality of 600 mOsmol l^− 1^ for 4, 8 and 24 h. After the hypertonic treatment, total RNA of gill filaments were extracted using mirVana miRNA isolation kit (Invitrogen) according to the manufacturer’s instruction. RNA quality was assessed using the Agilent 2100 Bioanalyzer system and samples with a RNA Integrity Number (RIN) greater than 8 was used for small RNA and RNA library constructions.

### Small RNA sequencing

Twenty small RNA libraries were constructed [five replicates per individual time points (4 and 8 h) per individual osmotic conditions (300 and 600 mOsmol l^− 1^)] using 5 μg of total RNA from each sample. Short RNA transcripts (18–30 nucleotides long) were resolved and isolated using Polyacrylamide gel electrophoresis (PAGE) gels. The selected small RNA molecules were ligated with 3′ and 5′ adapters, followed by the synthesis of single stranded complementary DNA (cDNA) using SuperScript II Reverse Transcriptase (Invitrogen). The cDNA was then amplified using indexing primers. After quantitative real-time PCR (qRT-PCR) amplification, the library size was determined using the Bioanalyzer (Agilent Technologies 2100). Concentrations of the libraries were tested using qRT-PCR (EvaGreen). The libraries were then processed by the Beijing Genomics Institute (BGI). Single-end read (50 bases pair (bp) read-length) was sequenced using the BGISEQ-500RS sequencer to produce at least 25 M clean reads per sample. Sequencing statistic was shown in Additional file 1: ST1. The adaptor sequences were trimmed and Phred quality score less than 20 were removed. The sequencing reads were mapped to the *Anguilla japonica* reference genome from NCBI (AVPY00000000.1, https://www.ncbi.nlm.nih.gov/Traces/wgs/?val=AVPY01) using mapper.pl module of the mirDeep2 package [52]. MicroRNAs were identified using the miRDeep2 procedure and final results were annotated for sequence similarity to known miRNAs in miRBase databases [53]. The quantification results produced by the quantifier.pl module of the mirDeep2 package were then used to compare the expression of miRNA in the different time points with the DESeq2 [54].

### Transcriptome sequencing and miRNA target genes prediction

Total RNA extracted from the same set of gill filament samples were subjected for cDNA library construction. The libraries were processed by BGI. Single-end reads, 50 bp read-length, were sequenced on the BGISEQ-500RS sequencer produce at least 25 M clean reads per sample. Sequence-reads were in turn trimmed according to BWA’s – q algorithm [55] and the sequence reads were quantified using the *Anguilla japonica* transcriptome assembly (Kallisto version 0.43.0) [56, 57]. Read-count data were then subjected to differential expression analysis using edgeR package [58]. Genes with |log2 (fold change: treatment/control)| > 0.5 and *q*-value < 0.01 were considered as differentially expressed genes (DEGs). Furthermore, Ingenuity Pathway Analysis (IPA®, QIAGEN Redwood City, www.qiagen.com/ingenuity) was used to decipher the effect of hypertonic treatment in gill filament. In the bioinformatics analysis, all the pathways, diseases or biofunctions with *P* < 0.05 were considered as statistically significant.

The miRanda algorithm [59] was used to predict genes targeted the miRNAs as previously described [60]. Briefly, the whole length of the miRNA was first aligned against the mRNA sequences. Thermodynamic stability of the RNA duplexes based on these alignments was calculated. Strict alignment in the seed region at position 2–7 bp of the miRNA to the mRNA sequence is required, of which the region is the key determinant in miRNA–mRNA recognition [61]. The target genes were then subjected to Database for Annotation, Visualization and Integrated Discovery (DAVID) v6.7 [62] to understand the functional roles of hypertonic treatment-deregulated miRNAs.

### Real-time PCR analysis of identified miRNAs and differential gene expression under hyperosmotic stress

Gill filaments were exposed to hyperosmotic treatment (600 mOsmol l^− 1^). After 4 h or 8 h incubation, total RNA of gill filaments were extracted using mirVana miRNA isolation kit (Invitrogen). For mature miRNA detection and quantification, 250 ng of total RNA was reverse transcribed in HiSpec buffer with miScript II RT kit (Qiagen). The cDNA generated is used for RT-PCR with miScript SYBR green PCR kit (Qiagen). Human small nuclear RNA 6 (RNU 6) (Qiagen: MS00033740) was used for normalizing RT-PCR of miRNA. Sequences for primers targeting miR-29b-3p and miR-200b-3p are miR-29b-3p: 5′- UAGCACCAUUUGAAAUCAGUGU-3′; miR-200b-3p: 5′-UAAUACUGCCUGGUAAU *GAUG*-3′. For differential gene expression analysis, cDNA generated with SuperScript VILO master mix (Invitrogen) was used for qRT-PCR analysis on hyper-osmotic and hypo-osmotic inducible genes. Sequences for specific primers targeting Japanese eel genes are shown in Additional file 1: ST2. Relative quantification of miRNA and mRNA expression was calculated based on delta delta Ct (ΔΔCt) values [57].

### Inhibition of miR activity

For experiments of miR-inhibition (miRi), primary gill cells were transfected with either mirVana™ miRNA inhibitor negative control #1 (Thermo Fisher, Cat #4464076) or mirVana™ target miRNA inhibitors (Thermo Fisher) at 100 nM in L-15 culture medium using Lipofectamine 2000 according to the manufacturer’s instructions (Invitrogen). After 24 h treatment, total RNA of miR-transfected cells was harvested for transcriptome sequencing. Quantitative RT-PCR was performed to assess the efficiency of inhibition. Sequences for miRNA inhibitors targeting miR-29b-3p, and miR-200-3p are 5′-UAGCACC AUUUGAAAUCAGUGU-3′ (Thermo Fisher, Assay ID: MH10432) and 5′-UAAUACUGC CUGGUAAUGAUG-3′ (Thermo Fisher, Assay ID: MH11741), respectively.

### Statistical analysis

Statistical analyses of DEGs and differentially expressed miRNA profiles were performed using GraphPad Prism 6 (GraphPad Software, San Diego, CA). Student’s t-test was used to assess for potential differences between control (isotonic) and treatment (hypertonic) groups. We reported statistical significance at *P < 0.05, **P < 0.01 and ***P < 0.001. Values were expressed as mean ± S.E.M.

## Supplementary information

**Additional file 1: Table S1.** Clean read stats: Small RNA sequencing statistic; **Table S2.** Primer sequence for RT-PCR: List of primer used for Real-time PCR analysis. **Table S3.** mRNA seq-4 h hyper: List of DEGs identified in eel gill filament culture under 4 h hypertonic stress treatment. **Table S4.** mRNAseq-8 h hyper: List of DEGs in eel gill filament culture under 8 h hypertonic stress treatment. **Table S5.** mRNAseq-miR-29b inhibition. List of upregulated genes in primary gill cell culture, after miR-29b-3p inhibition. **Table S6.** mRNAseq-miR-200b inhibition: List of upregulated genes in primary gill cell culture, after miR-200b-3p inhibition.

## Data Availability

The datasets supporting the conclusions of this article are included within the article and its additional files.

## Abbreviations

aqp-3: Aquaporin-3
at1a1: ATPase Na^+^/K^+^ transporting subunit a1
at1a3: ATPase Na^+^/K^+^ transporting subunit a3
at2b2: Calcium-transporting ATPase 2
ATP: Adenosine triphosphate
ATPases: Adenosine triphosphatases
BGI: Beijing Genomics Institute
bp: Bases pair
CCs: Chloride cells
cDNA: Complementary DNA
cftr: Cystic fibrosis transmembrane regulator
DEGs: Differentially expressed genes
dicer-1: Dicer ribonuclease III
drosha: Drosha RNase III endonuclease
FBS: Fetal bovine serum
FW: Freshwater
GH: Growth Hormone
IGF: Insulin-like growth factor
Kir: Inward rectifying K^+^ channel
Klf4: Kruppel-like factor 4
L-15: Leibovitz’s L-15
miR: MicroRNA
Nhe: Na^+^/H^+^ exchanger
nherf1: Na^+^/K^+^-exchanger regulatory cofactor
Nkcc, s12a2: Na^+^/K^+^/2Cl^−^ cotransporter
orebp: Osmotic response element binding protein
ostf1: Osmoregulatory transcription factor
PAGE: Polyacrylamide gel electrophoresis
Pri-miR: Primary-miR
PS: Penicillin-streptomycin
PVC: Pavement cells
RIN: RNA Integrity Number
S17a5, Slc17a5: Solute carrier family 17 member 5
SW: Seawater
taut: Na^+^/Cl^−^-taurine transporter
UTR: Untranslated region
vpp1: V-type proton ATPase 1
wnk1: With-no-lysine kinase
xpo5: Exportin-5
